# Redox-Controlled Chemical Protein Synthesis: Sundry Shades of Latency

**DOI:** 10.1021/acs.accounts.2c00436

**Published:** 2022-09-09

**Authors:** Vangelis Agouridas, Nathalie Ollivier, Jérôme Vicogne, Vincent Diemer, Oleg Melnyk

**Affiliations:** † Univ. Lille, CNRS, Inserm, CHU Lille, Institut Pasteur de Lille, U1019-UMR 9017, Center for Infection and Immunity of Lille, F-59000 Lille, France; ‡ Centrale Lille, F-59000 Lille, France

## Abstract

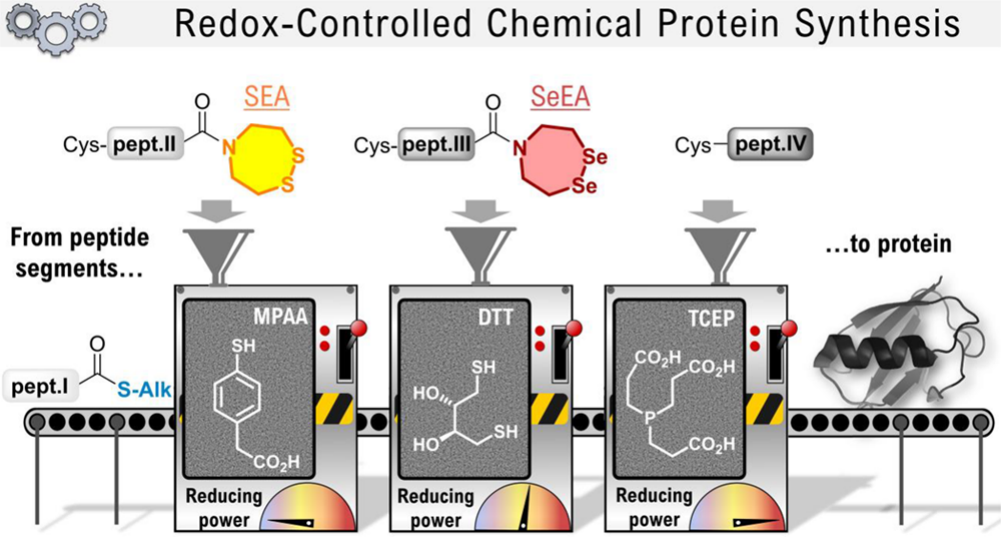

The last two decades have witnessed the rise in power of chemical protein synthesis to the point where it now constitutes an established corpus of synthetic methods efficiently complementing biological approaches. One factor explaining this spectacular evolution is the emergence of a new class of chemoselective reactions enabling the formation of native peptide bonds between two unprotected peptidic segments, also known as native ligation reactions. In recent years, their application has fueled the production of homogeneous batches of large and highly decorated protein targets with a control of their composition at the atomic level. In doing so, native ligation reactions have provided the means for successful applications in chemical biology, medicinal chemistry, materials science, and nanotechnology research.

The native chemical ligation (NCL) reaction has had a major impact on the field by enabling the chemoselective formation of a native peptide bond between a C-terminal peptidyl thioester and an N-terminal cysteinyl peptide. Since its introduction in 1994, the NCL reaction has been made the object of significant improvements and its scope and limitations have been thoroughly investigated. Furthermore, the diversification of peptide segment assembly strategies has been essential to access proteins of increasing complexity and has had to overcome the challenge of controlling the reactivity of ligation partners.

One hallmark of NCL is its dependency on thiol reactivity, including for its catalysis. While Nature constantly plays with the redox properties of biological thiols for the regulation of numerous biochemical pathways, such a control of reactivity is challenging to achieve in synthetic organic chemistry and, in particular, for those methods used for assembling peptide segments by chemical ligation. This Account covers the studies conducted by our group in this area. A leading theme of our research has been the conception of controllable acyl donors and cysteine surrogates that place the chemoselective formation of amide bonds by NCL-like reactions under the control of dichalcogenide-based redox systems. The dependency of the redox potential of dichalcogenide bonds on the nature of the chalcogenides involved (S, Se) has appeared as a powerful means for diversifying the systems, while allowing their sequential activation for protein synthesis. Such a control of reactivity mediated by the addition of harmless redox additives has greatly facilitated the modular and efficient preparation of multiple targets of biological relevance. Taken together, these endeavors provide a practical and robust set of methods to address synthetic challenges in chemical protein synthesis.

## Key References

- Raibaut, L.; Cargoët, M.; Ollivier, N.; Chang, Y. M.; Drobecq, H.; Boll, E.; Desmet, R.; Monbaliu, J.-C. M.; Melnyk, O. Accelerating Chemoselective Peptide Bond Formation Using *bis*(2-Selenylethyl) Amido Peptide Selenoester Surrogates. Chem. Sci.2016, 7, 2657–26652866003810.1039/c5sc03459kPMC5477010.^1^ This study discusses in detail the concepts related to the latency of *bis*(2-sulfanylethyl)amido (SEA) and *bis*(2-selanylethyl)amido (SeEA) thioester surrogates, their acyl donor potencies, and how redox and kinetically controlled assembly processes can be combined for the total synthesis of the biotinylated NK1 protein, a 20 kDa protein derived from the hepatocyte growth factor (HGF).
- Ollivier, N.; Desmet, R.; Drobecq, H.; Blanpain, A.; Boll, E.; Leclercq, B.; Mougel, A.; Vicogne, J.; Melnyk, O. A Simple and Traceless Solid Phase Method Simplifies the Assembly of Large Peptides and the Access to Challenging Proteins. Chem. Sci.2017, 8, 5362–53702897091510.1039/c7sc01912bPMC5609153.^2^ This article is a useful complement to this Account by discussing the application of SEA latent thioester chemistry to the synthesis of proteins through iterative ligation on a water-compatible solid support.
- Diemer, V.; Ollivier, N.; Leclercq, B.; Drobecq, H.; Vicogne, J.; Agouridas, V.; Melnyk, O. A Cysteine Selenosulfide Redox Switch for Protein Chemical Synthesis. Nat. Commun.2020, 11, 2558.3244476910.1038/s41467-020-16359-6PMC7244499(3) This study shows that *N*-(2-selanylethyl)cysteine (SetCys) is a redox-controlled cysteine surrogate that can be combined with the SEA group for the one-pot synthesis of cyclic proteins. This article also provides a robust kinetic model for SetCys-mediated ligation.
- Snella, B.; Grain, B.; Vicogne, J.; Capet, F.; Wiltschi, B.; Melnyk, O.; Agouridas, V. Fast Protein Modification in the Nanomolar Concentration Range Using an Oxalyl Amide as Latent Thioester. Angew. Chem. Int. Chem.2022, e20220499210.1002/anie.20220499235557487.^4^ This study describes the capacity of *bis*(2-sulfanylethyl) oxalamides (oxoSEA) to act as an efficient acyl donor in the nanomolar concentration range in purified or complex media such as cell lysates.

## 1 Introduction

**Figure 1:**
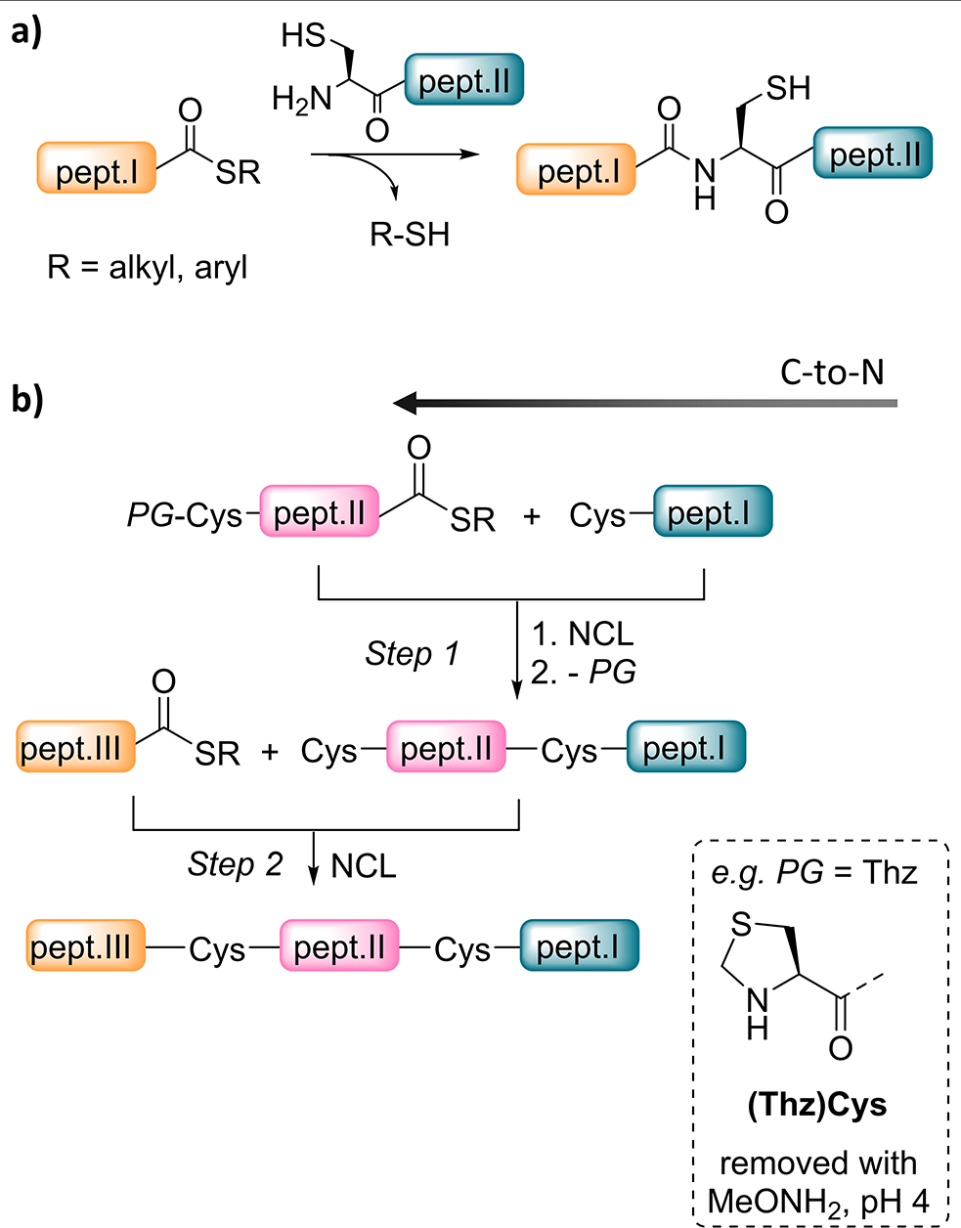
Native chemical ligation reaction. (a) Principle. (b) NCL-mediated peptide segment assembly using a Cys protecting group strategy.

“To equal Nature...I therefore foresee the day when physiological chemistry will not only make extensive use of the natural enzymes as agents, but when it will also prepare synthetic ferments for its purposes” said Emil Fischer in his Nobel Lecture in 1902.^5^ Emil Fischer’s prediction came true in 1969 when Bruce Merrifield could produce a fully synthetic and functional ribonuclease A enzyme from component amino acids using the solid phase peptide synthesis (SPPS).^6^ Since then, chemical protein synthesis has become an essential method for the study of protein structure and function, for the investigation of cellular mechanisms, or for the development of therapeutic molecules due to its capacity to access proteins with virtually any kind of modification or functional probe. Since the synthesis of ribonuclease A enzyme, the methods used for accessing proteins by chemical synthesis have considerably evolved. While pioneering works privileged the SPPS based on the stepwise coupling of protected amino acids in organic solvents,^7^ modern chemical protein synthesis primarily relies on the chemoselective coupling of unprotected peptide segments in aqueous media among which the native chemical ligation (NCL^8^) or the ketoacid-hydroxylamine (KAHA^9^) or serine/threonine (STL^10^) ligations are more advanced. The assembly of proteins from shorter peptide segments is faced with the diversity of the peptide junctions that has to be made and with the variable solubility and stability of the peptide segments that are linked together. Tremendous efforts are devoted to solve these issues especially by extending the scope of the NCL reaction, the most widely used peptide ligation method for chemical protein synthesis up to now (Figure 1a).^11^ NCL relies on the coupling of a peptide thioester with a cysteinyl peptide to produce a longer peptide with a novel peptide bond to cysteine. The performance of chemical protein synthesis using NCL can be appreciated by the number of functional proteins produced so far as well as the exceptional size of some synthetic proteins accessed through this chemistry.^12,13^

Nevertheless, the future of chemical protein synthesis and its spread to the chemical biology community largely depends on the ease with which a protein can be assembled from individual peptide segments. While peptides can be routinely produced using SPPS and automated protocols, this is not yet the case for proteins, but the field is clearly advancing in this direction.^14^ We believe that a key to success resides in the simplification of the peptide segment assembly strategies. This was the main goal of our research efforts during the past decade. Rather than make us go back, the search for simplicity required innovation and led us to the conception of novel redox-controlled ligation chemistries related to NCL.

## 2 Chemical Protein Synthesis: The Search for Pot-Economy

The assembly of a protein from a collection of shorter peptide segments is inevitably confronted with the need to sequentially mask and unmask the reactivity of chemical groups involved in the formation of the peptide bonds as the synthesis progresses. In the example provided in Figure 1b, a protein is assembled from three peptide segments by elongating the peptide chain in the C-to-N direction using NCL. This strategy implies the presence of two N-terminal Cys residues at step 1. Although NCL is highly chemoselective, it cannot distinguish between two Cys residues that are freely accessible. One classical and very popular approach to direct the peptide segment assembly in such a case is to equip the N-terminal Cys residue of the middle peptide segment with a temporary protecting group (PG), which must be removed before running the second ligation step.

The mean size of the peptide segments used for protein chemical synthesis is in average less than 40 AAs, a value that reflects the performance of the SPPS.^13^ Therefore, the access to proteins of larger size will require a higher number of peptide segments to be concatenated and, thus, will result in an increase of process complexity due to the accumulation of chemical steps and intermediate purifications, whatever the chemical strategy used to control the protein assembly. Consequently, simplification of protein chemical synthesis has been early viewed as a critical point to address for accessing proteins of exceptional size. In that, protein chemists are confronted with issues that have been experienced by organic chemists for a long time in their quest for the chemical synthesis of complex natural products. Today, the minimal use of protective groups and the integration of the dimensions of step-^15^ and pot-economy^16^ in process design is perceived as natural when it comes to envisage the synthesis of a protein, and such a tendency participates in making protein chemical synthesis more popular.

Proteins are a special kind of “natural products” in that their total synthesis requires manipulation and especially purification of polar polypeptide intermediates. Numerous reports have documented moderate or weak recoveries of large polypeptides when classical methods of chromatography such as reversed-phase HPLC are used.^17^ This is the reason that the performance of a series of chemical transformations in the same reactor has been the focus of so many research efforts. The development of one-pot peptide assembly methods started with the work of Bang and Kent on the synthesis of crambin using the thiazolidine protection for N-terminal cysteine in 2004 (Figure 1b).^18^ Such a use of Cys PGs for the design of one-pot peptide assembly strategies remains a timely and dynamic area of research considering the efficient approaches of this kind published recently.^11,19^

Inspired by the significant gain in synthetic efficiency provided by these one-pot approaches, we considered taking such a strategy a step further. Is it possible to make it more pot-economical without using Cys PGs to control the selectivity of the peptide assembly? This question took us in a conceptually different direction by placing the concept of thioester group latency at the heart of our work.

## 3 Latent Functional Groups: An Old Trick for New Challenges

**Figure 2:**
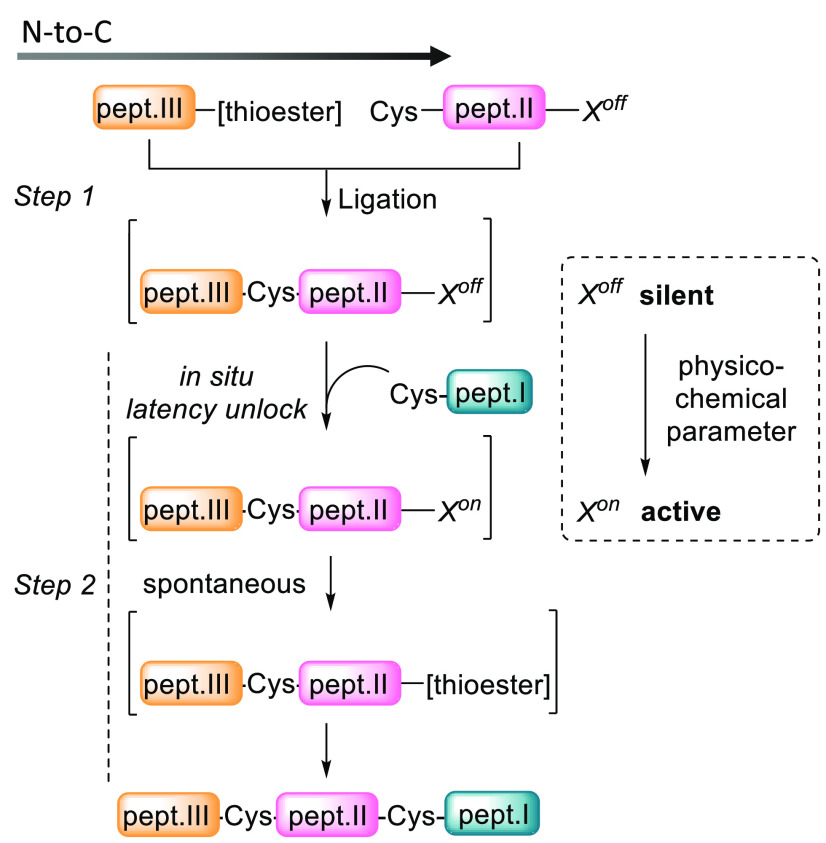
Concept of latency illustrated with the one-pot assembly of three peptide segments.

The concept of latent functionality was formalized for the first time by Lednicer in 1972^20^ and widely applied afterward.^21^ According to the definition given by Lednicer, “this idea, which we have chosen to call *latent functionality*, carries some necessary function through one or more steps of a synthesis in a precursor form; at the proper stage the precursor is converted to the needed group”. The decision to use latent thioesters instead of protected cysteines during the peptide segment assembly implies the elongation process should be reconfigured in the N-to-C direction as depicted in Figure 2. At Step 1 of the process, only the N-terminal Cys and the active thioester group present in the reaction mixture can react, the second thioester being masked under its latent form *X*^off^ during the ligation. The design of latent chemical systems, presenting two well-defined silent (*X*^off^) and active (*X*^on^) states is necessary. Otherwise, the residual reactivity expressed during the latency period would inevitably affect the selectivity of the assembly, and the yield of the desired ligation product would depend on the differential reactivity of the present acyl donors. This is typically what happens during kinetically controlled ligations (KCLs^22^), which also enable protein synthesis in the N-to-C direction. The concepts developed in this Account differ markedly from KCL approaches in that the second thioester functionality needed for Step 2 is generated only after the first ligation step is completed.

The need to make the peptide assembly more pot-economical led us to think how to place the latent functionality under the control of an easily-tunable physicochemical parameter of the aqueous solution where the protein assembly takes place. In response to the applied stimulus, the active thioester group would be generated *in situ* after a series of spontaneous chemical transformations.

Now comes the question of which physicochemical parameter to choose for the design of latent thioester systems. The use of pH as a parameter to control peptide assembly is limited by the narrow range of pH tolerated by NCL. Indeed, Cys thiolates are the nucleophilic species in NCL and are significantly populated only above pH 5.5–6,^23^ while thioesters are prone to hydrolysis in basic media. Consequently, reports using the pH to control protein assembly are rare.^24^ The sensitivity of some chemical systems to general acid or base catalysis gives opportunities to place the latent thioester under the control of the buffer concentration. This mode of control was studied by Otaka and co-workers with the SEAlide latent thioester system, which is responsive to phosphate buffer concentration.^25^ The use of temperature has great potential as it enables an external control of the process with no significant change of pH or other reaction parameters.^26^ However, the design of temperature-dependent chemical systems is a difficult task as it requires elevated activation energies to provide a strong response within a narrow range of temperature. An acceptable temperature range for reactions conducted with peptide segments in water is usually 10–55 °C, unless special setups such as microfluidic conditions are employed.^27^

**Figure 3:**
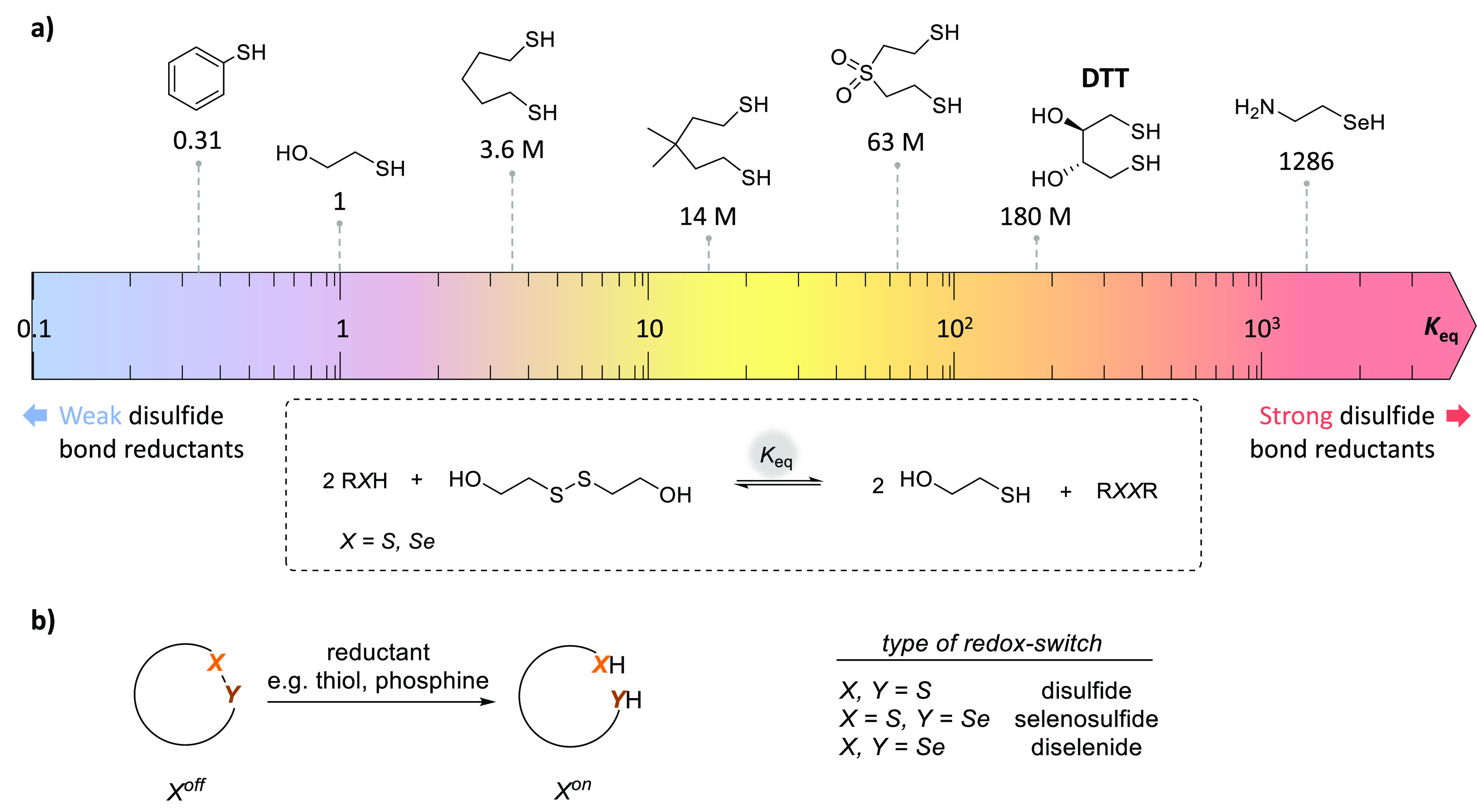
Sulfur and selenium chemistries enable the design of redox-switches. (a) Thiol–disulfide interchange equilibrium constant relative to mercaptoethanol (*K*_eq_) for a selection of thiols and selenocysteamine. (b) Redox-switches based on cyclic dichalcogenide structures.

In search for a physicochemical parameter that could strongly vary at neutral pH, we identified the very large spectrum of reducing powers attainable in water with simple and well-tolerated reagents such as thiols and phosphines. Indeed, just the consideration of the reducing power of thiols, appreciated through the value of the thiol–disulfide interchange equilibrium constant relative to mercaptoethanol disulfide, shows differences of several orders of magnitude by going from aromatic thiols to dithiols such as 1,4-dithiothreitol (DTT) (Figure 3a).^29^ We therefore chose to design redox-sensitive systems for peptide ligation though NCL that would have to accommodate the presence of thiols such as the Cys thiol involved in the NCL step and the monothiol catalysts classically used to promote the ligation.^28^ The high stability of certain cyclic disulfides toward aryl and alkyl thiol reductants, especially those formed from 1,4- and 1,5-dithiols, led us to propose cyclic disulfides as a means to place the latency under redox control (X, Y = S; Figure 3b). Knowing also the large difference in redox potential between disulfides and selenosulfides or diselenides, we perceived the opportunity to create a family of latent systems responding to incremental changes in reducing power by combining sulfur and selenium chemistries (X, Y = S, Se; Figure 3b).

## 4 Carboxamides as Redox-Controlled Latent Thioesters

**Figure 4:**
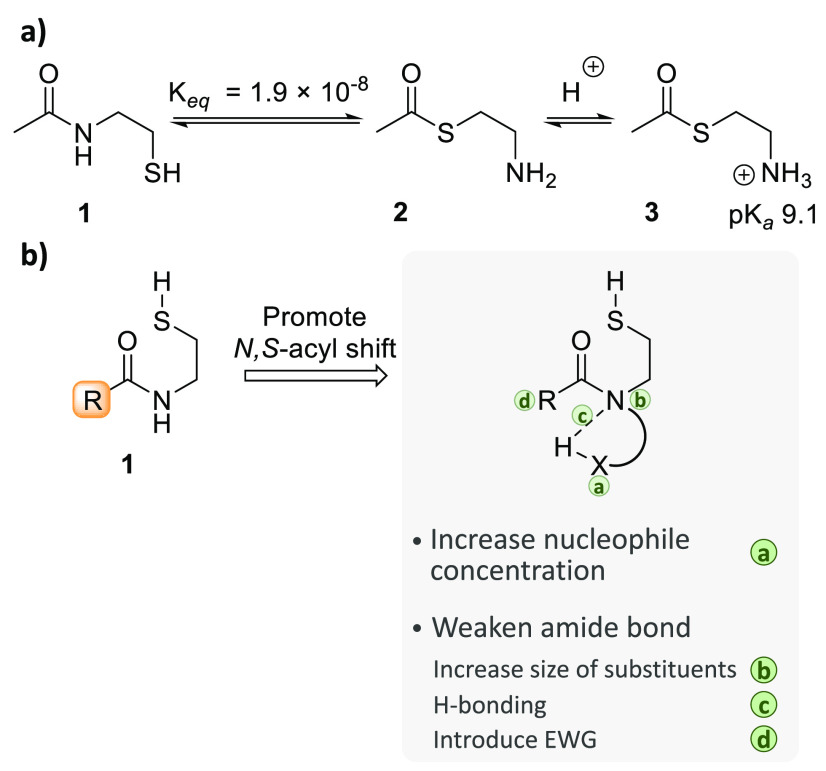
Rearrangement of *N*-(2-mercaptoethyl) amides into thioesters. (a) The case of *N*-acetylmercaptoethylamine **1**. (b) Possible means for favoring the *N*,*S*-acyl shift of *N*-(2-mercaptoethyl) amides.

Then came the time to know how to implement these ideas for the creation of redox-sensitive thioesters. Studies conducted by Barnett and Jencks in the late 1960’s on the rearrangement of *N*-acetylmercaptoethylamine **1** into *S*-acetylmercaptoethylamine **2**/**3** in aqueous acid was particularly inspiring (Figure 4a).^30^ In such an equilibrium, the thioester form **2**/**3** is significantly populated only in highly acidic media (pH < 2) due to the masking of the amine nucleophile upon protonation. Therefore, the conception of latent thioesters based on the natural capacity of *N*-(2-mercaptoethyl) amides of type **1** to equilibrate with thioesters of type **2**/**3** had to overcome a major obstacle in making the rearrangement happen at the working pH of NCL. The migration of the acyl group from nitrogen to sulfur can potentially be promoted by weakening the amide bond (Figure 4b). Previous works showed that this could be achieved by introducing electron-withdrawing groups (EWGs) alpha to the carbonyl,^31^ by increasing the size of the substituents on the amide nitrogen,^32^ or by enabling intramolecular hydrogen bonding to the amide nitrogen.^33^ The latter strategy can be particularly powerful as shown by the impact of a weak intramolecular hydrogen bond to the prolyl nitrogen on the barrier of amide isomerization.^33^ Another means to favor the rearrangement of amides of type **1** is to increase the concentration of the nucleophile that attacks the amide carbonyl (Figure 4b).

**Figure 5:**
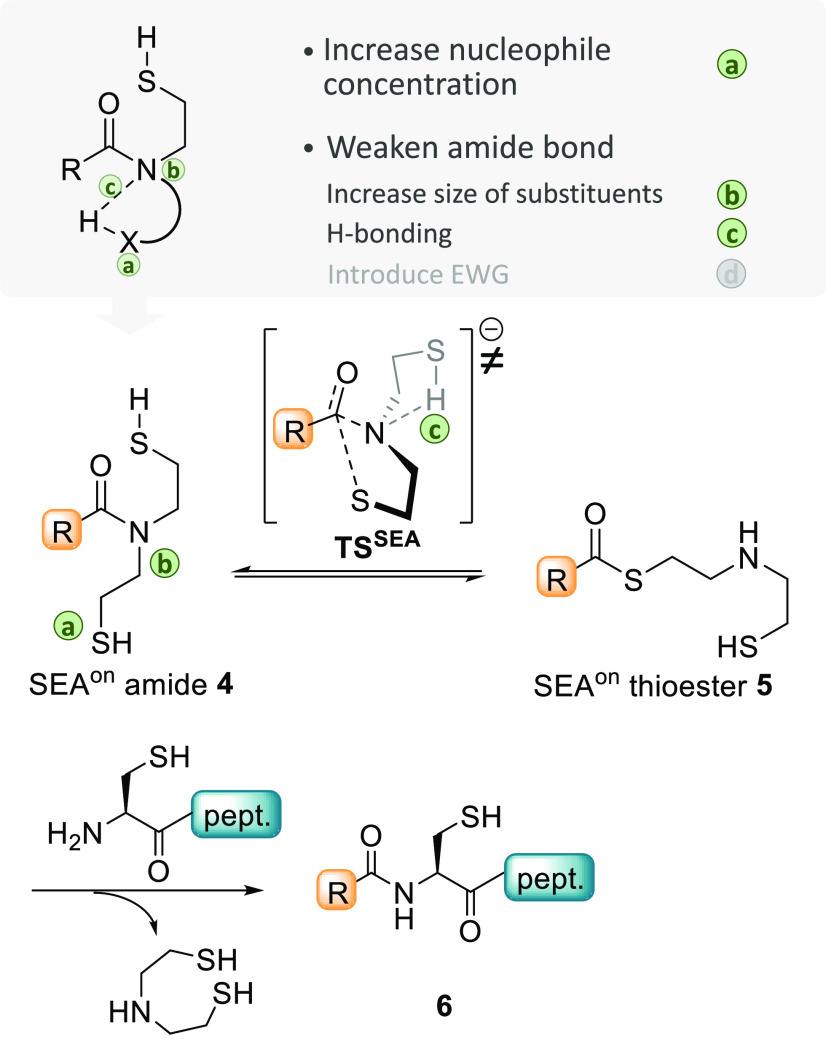
Conception of the *bis*(2-sulfanylethyl)amido (SEA) system.

It turns out that simply appending a second 2-mercaptoethyl limb to the amide nitrogen of **1** yields the *bis*(2-sulfanylethyl) amide (SEA) system **4**, which can potentially benefit from several of the activation mechanisms discussed above (Figure 5). Indeed, doing so increases the size of amide nitrogen substituents and the number of thiol groups around the amide carbonyl and creates a favorable arrangement for an intramolecular H-bonding to the amide carbonyl to occur. Experimentally, SEA amides of type **4** act as thioester surrogates at slightly acidic or neutral pH and react with N-terminal cysteinyl peptides to produce a peptide bond to cysteine.^34^ This reactivity contrasts with that of monovalent *N*-(2-mercaptoethyl) amides of type **1** or *N*,*N*-(2-mercaptoethyl) alkyl amides lacking the second thiol group. Experimentations using SEA peptides showed that SEA-mediated reactions are usually under the kinetic control of the *N*,*S*-acyl shift process (**4** → **5** in Figure 5, R = peptide).^1^ Computational studies using H–CO–Gly–N(CH_2_CH_2_SH)_2_ (R = H–(C=O)–NHCH_2_– in Figure 5) as a model for SEA peptides showed an intramolecular S–H–N interaction in the transition state **TS**^**SEA**^ of lowest energy. As the calculations suggest, the SEA amide system **4** enables one thiolate to act as the nucleophile while the other thiol transfers its proton to the amide nitrogen in the transition state. The exact nature of the *N*,*S*-acyl shift transition state remains to be established, probably by conducting more in depth computational studies that take into account explicit water molecules. Regarding the scope and limitations of the SEA-mediated ligation, we noticed that SEA-peptide epimerization or hydrolysis is usually insignificant during ligation, which proceeds optimally at pH 5.5 under an inert atmosphere.^1^ The rate of SEA-mediated ligation and occurrence of side reactions is impacted by the nature of the C-terminal amino acid bearing the SEA functionality in a manner that follows the behavior of classical peptide alkyl thioesters.^11^

**Figure 6:**
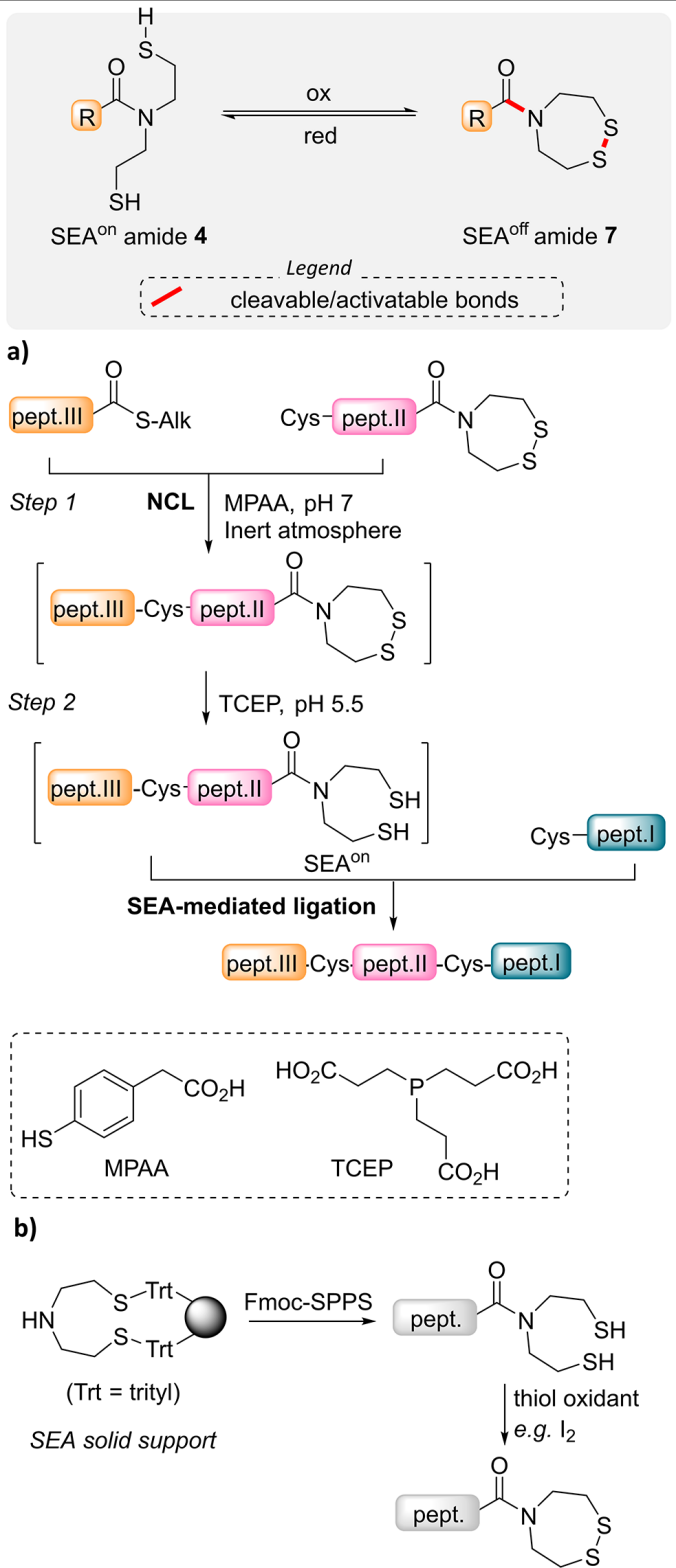
SEA^off^ latent thioester. (a) Sequential NCL and SEA-mediated ligations enable the redox-controlled assembly of three peptide segments in one-pot. (b) Fmoc-SPPS of SEA^off^ peptides.

A second important consequence of appending a second 2-mercaptoethyl limb to the amide nitrogen of **1** is to create a 1,5-dithiol structure in amide **4**, a feature that enables one to place the SEA acyl donor under redox control (Figure 6). The cyclic form **7**, which is formally an *N*-acyl-1,2,5-dithiazepane,^35^ is called SEA^off^. The SEA^off^ amide is extremely stable toward various reagents such as acids, bases, and amine nucleophiles as can be expected for a tertiary carboxamide. Such a property facilitates the installation of a SEA amide group into peptides using standard solid phase or solution protocols.^4,36^ Also, and as anticipated from the thiol–disulfide interchange equilibrium constants presented in Figure 3a, SEA^off^ amide **7** is stable in the presence of large excesses of 4-mercaptophenylacetic acid (MPAA), the gold-standard catalyst of NCL, and under conditions that enable the reduction of acyclic disulfides, especially those derived from cysteine. Hence, SEA^off^ amide **7** does not interfere with the NCL reaction using regular thioester components, provided strong reductants are excluded from the ligation mixture. In contrast, the reduction of the cyclic disulfide bond using DTT or *tris*(2-carboxyethyl)phosphine (TCEP) unlocks the acyl donor capabilities of the SEA system by triggering the *N*,*S*-acyl shift and the formation of thioester **5** from amide **4**. This binary chemical behavior makes the assembly of three peptide segments in one-pot under redox control extremely simple to setup (Figure 6a). In the first step, a regular NCL reaction is performed in absence of a strong reductant under an inert atmosphere, conditions that enable one to keep the SEA^off^ group inactive while minimizing thiol oxidation by molecular oxygen. Note that, under such conditions, the partial formation of mixed disulfides between Cys thiols and MPAA is frequently seen without affecting the ligation outcome. Then, the addition of TCEP and the third segment triggers the second ligation step and the formation of the full-length polypeptide.

The process described in Figure 6a provides a straightforward access to proteins made of 100–150 amino acids, if individual peptide segments are produced by SPPS. For comparison, the size of the protein domains observed in 3D-structure databases shows a narrow distribution with a maximum frequency of around 100–150 amino acids.^37^ Therefore, such a three peptide segment assembly process covers many needs in chemical biology programs. It can be set up with ease considering the facility to access SEA^off^ peptides by 9-fluorenylmethyloxycarbonyl (Fmoc) SPPS using SEA solid supports (Figure 6b).^34^ Very recently, Kajihara and co-workers established a means for accessing C-terminal SEA^off^ polypeptides from recombinant precursors produced in *E. coli*.^38^ Therefore, the redox-controlled assembly of large proteins according to Figure 6 is now potentially feasible using peptide segments of extended length produced in live cells. An alternative to access large protein targets consists of an extension of the principles of the solution process depicted in Figure 6 to a water-compatible solid support for the stepwise concatenation of peptide segments.^2,39,40^ Doing so pushes the concept of pot-economy one step further and provides a potential solution to automated chemical protein synthesis.^14^

**Figure 7:**
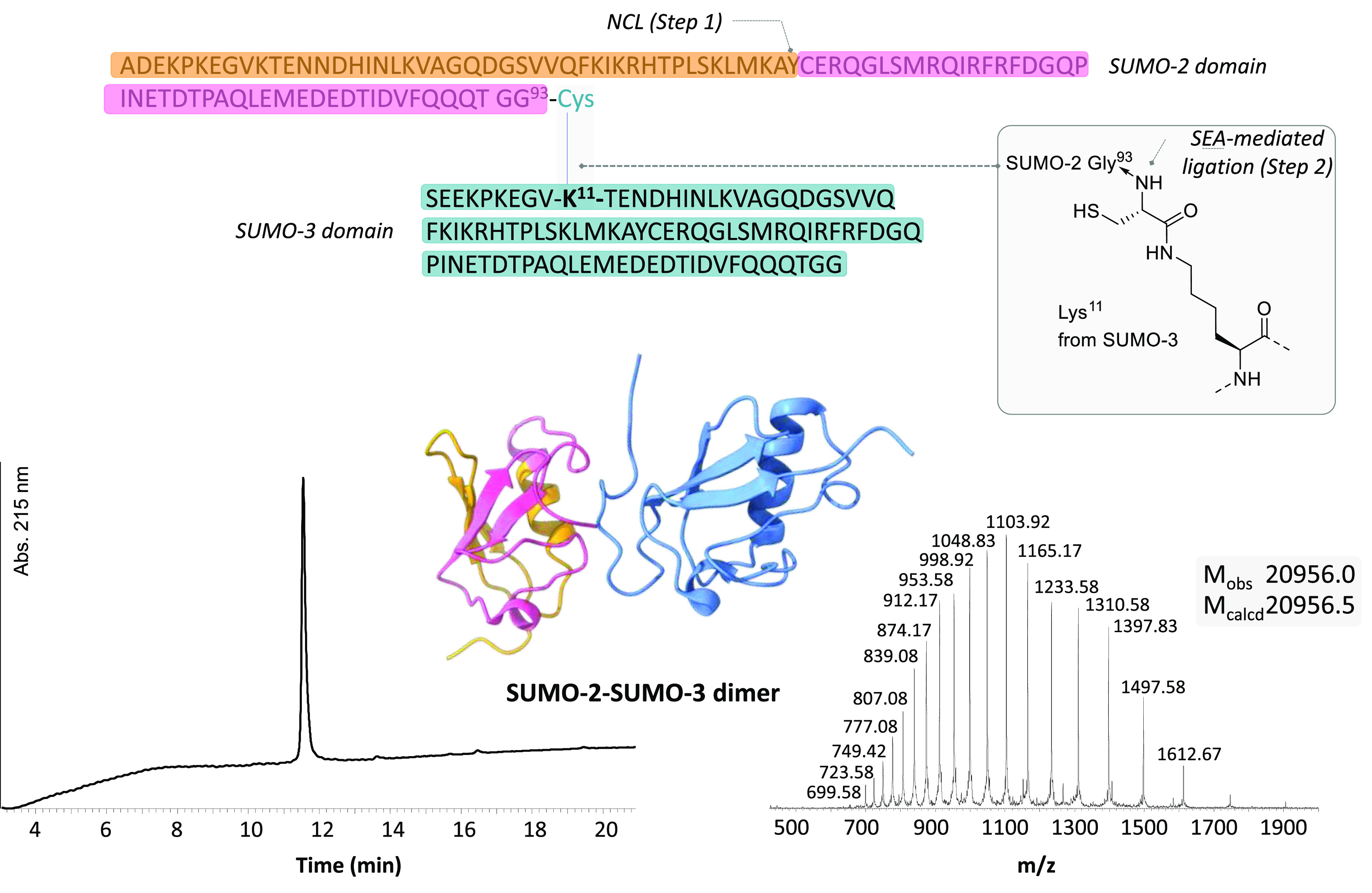
Total synthesis of a SUMO-2-SUMO-3 dimer protein analog (adapted from PDB 2mp2). The color code corresponds to the ligated peptide segments; see Figure 6.

The performance of the one-pot redox-controlled three peptide segment assembly method can be appreciated with the total synthesis of SUMO proteins, a type of post-translational modification related to ubiquitin.^41−43^ The synthesis of a SUMO-2-SUMO-3 dimer protein analog having a size of 21 kDa is described in Figure 7.^41^ The assembly of the SUMO-2 domain was performed at Step 1 using a cysteine residue naturally present in its central position. A cysteine residue was introduced on the side chain of Lys^11^ within the SUMO-3 domain to facilitate the formation of a branched product upon SEA-mediated ligation with the SUMO-2 domain obtained from the previous step.

**Figure 8:**
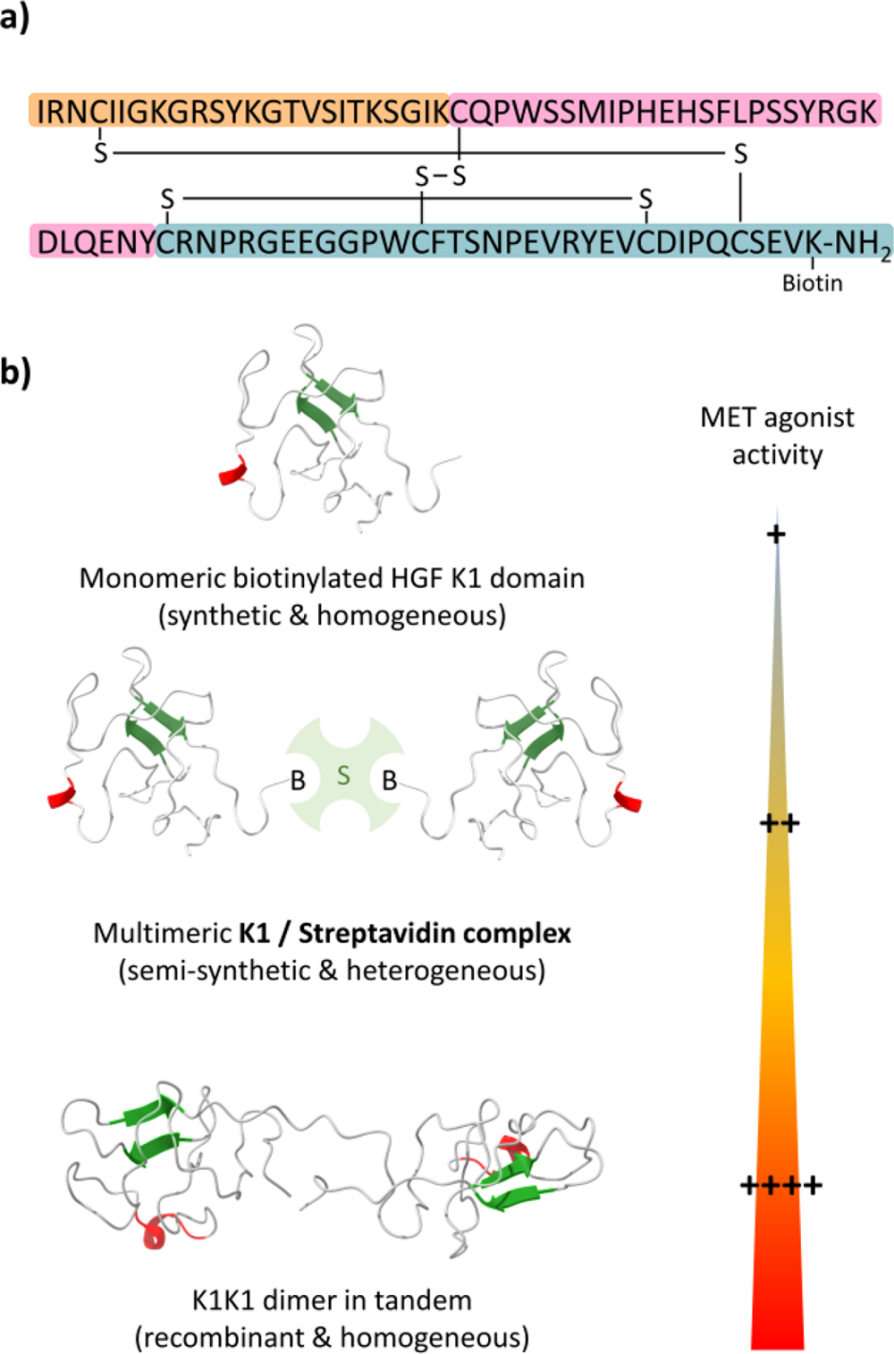
From the chemical synthesis of the biotinylated HGF K1 domain to the design of a potent recombinant MET agonist made of two K1 domains in tandem. (a) Sequence of the biotinylated K1 domain. The color code corresponds to the ligated peptide segments; see Figure 6. (b) MET agonist activity of K1 constructs shows a strong dependence on multivalency (K1: PDB 1bht; K1K1dimer: PDB 7ocl).

The second application we want to discuss is a concrete illustration of the power of chemical protein synthesis for studying protein function. It had to overcome the challenge of folding proteins stabilized by multiple disulfide bonds, a problem to which protein chemists are frequently confronted after having assembled the linear polypeptide precursor. In our case, we have achieved native folds in good yields by using redox glutathione buffers. Note that the understanding of protein folding and the development of methods enabling a full control of the disulfide bond pattern upon folding is a timely topic with recent remarkable achievements.^44^ In the work summarized in Figure 8, we used SEA chemistry to produce milligram quantities of the biotinylated kringle 1 (K1) domain from the hepatocyte growth factor (HGF). This synthetic protein enabled one to clarify the role of the K1 domain in the binding and activation of the MET tyrosine kinase receptor. In particular, a biotin label was installed on the protein to investigate the role of multivalency in the agonistic activity of the K1 domain using streptavidin (S) as a presentation platform. Contrary to the monomeric K1 domain, semisynthetic K1/streptavidin mixtures of complexes containing (K1)_2_S as the major component displayed strong agonistic activities.^45^ This observation motivated the design of a recombinant covalent K1K1 dimer molecule linking two K1 domains in tandem, which displays even stronger MET agonist activity *in vitro* and *in vivo*.^46^

**Figure 9:**
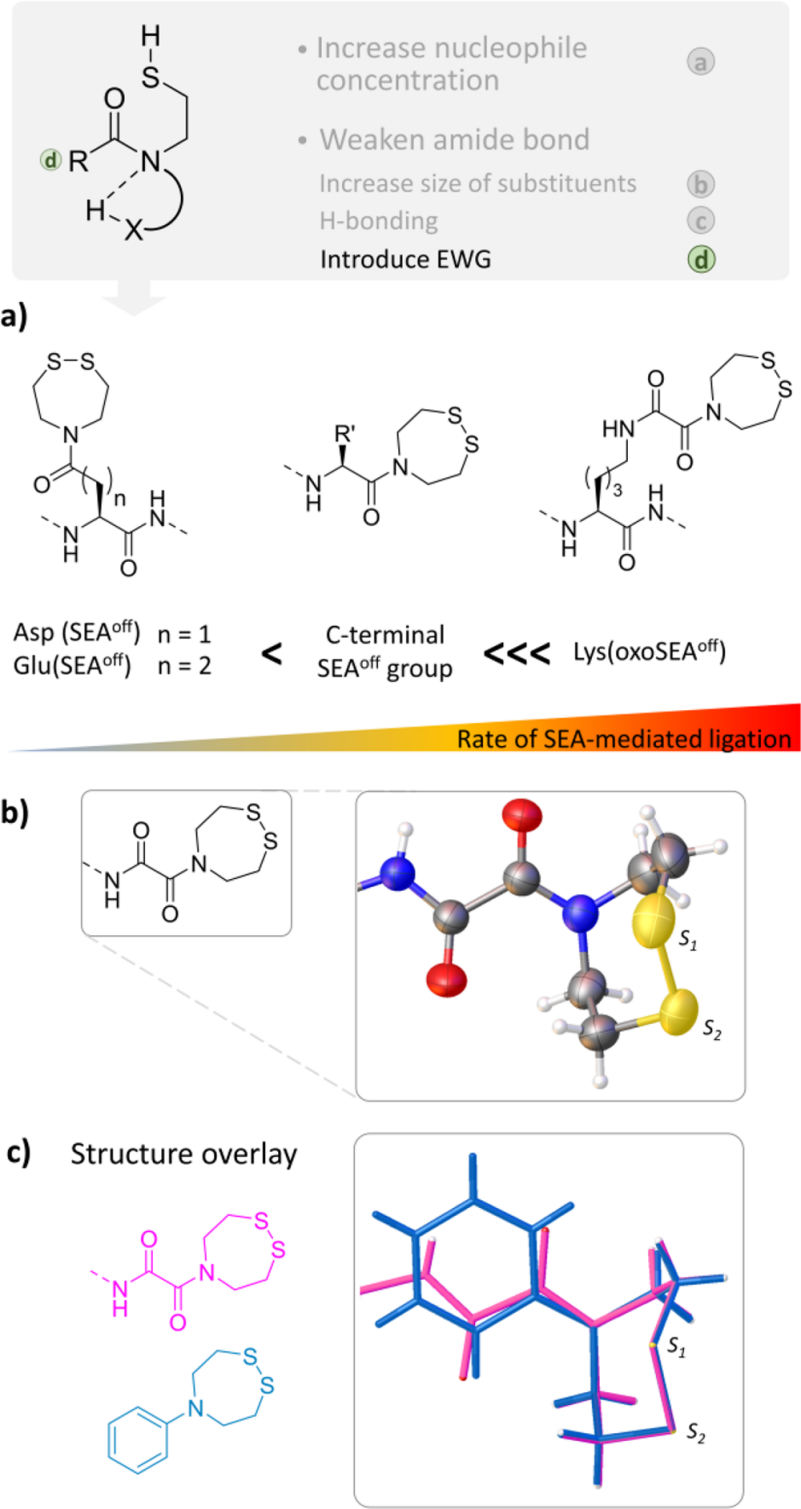
Rate of SEA-mediated ligation increases with the electron withdrawing capability of the R group linked to the SEA group carbonyl. (a) Relative acyl donor capabilities for different SEA systems. (b) X-ray structure of the oxalyl SEA group.^4^ (c) The SEA 1,2,5-dithiazepane heterocycle adopts a chairlike conformation,^4^ similar to that described for 5-phenyl-1,2,5-dithiazepane.^47^

One hallmark of the SEA^off^ system is to keep its latent properties regardless of the nature of the R group linked to the SEA amide carbonyl (Figure 9a). In contrast, the reactivity of the SEA^on^ system is dramatically affected by the electron withdrawing capability of the R group. These features enable us to significantly extend the scope of SEA chemistry. For example, the SEA^off^ group can be easily installed on aspartic or glutamic acid side chains and serve as a ligation site to access elaborated peptide scaffolds.^36^ The deportation of the SEA group from the C-terminal position to the side chain of Asp/Glu is at the price of a reduction of the acyl donor capability due to the increased distance between the electron withdrawing alpha nitrogen and the SEA carbonyl in the Asp/Glu(SEA) systems. Oppositely, the introduction of a strong electron withdrawing group in R as in the oxalyl SEA (oxoSEA) system enabled us to achieve exceptional rates approaching 30 M^–1^ s^–1^, a property that permitted ligation to proceed in the nanomolar range in purified or complex media such a cell lysates.^4^

To sum up at this stage, the SEA^off^ amide group discussed above enriches the growing family of latent thioester systems, which include peptide hydrazides^48,49^ and some *N*,*S*-acyl shift systems^50,51^ such as protected cysteinyl prolyl esters^52^ or photocaged SEAlide.^53^ However, at this stage, the SEA^off^ group appears as a single shot rifle enabling no more than three peptide segments to be assembled in one-pot (see Figure 2). As a logical continuation of our work, we wondered if it would be possible to assemble more than three peptide segments in one-pot under redox control? The answer to this question is yes, and the next section describes how we an achieved such a goal.

## 5 Sea: From Sulfur to Selenium

**Figure 10:**
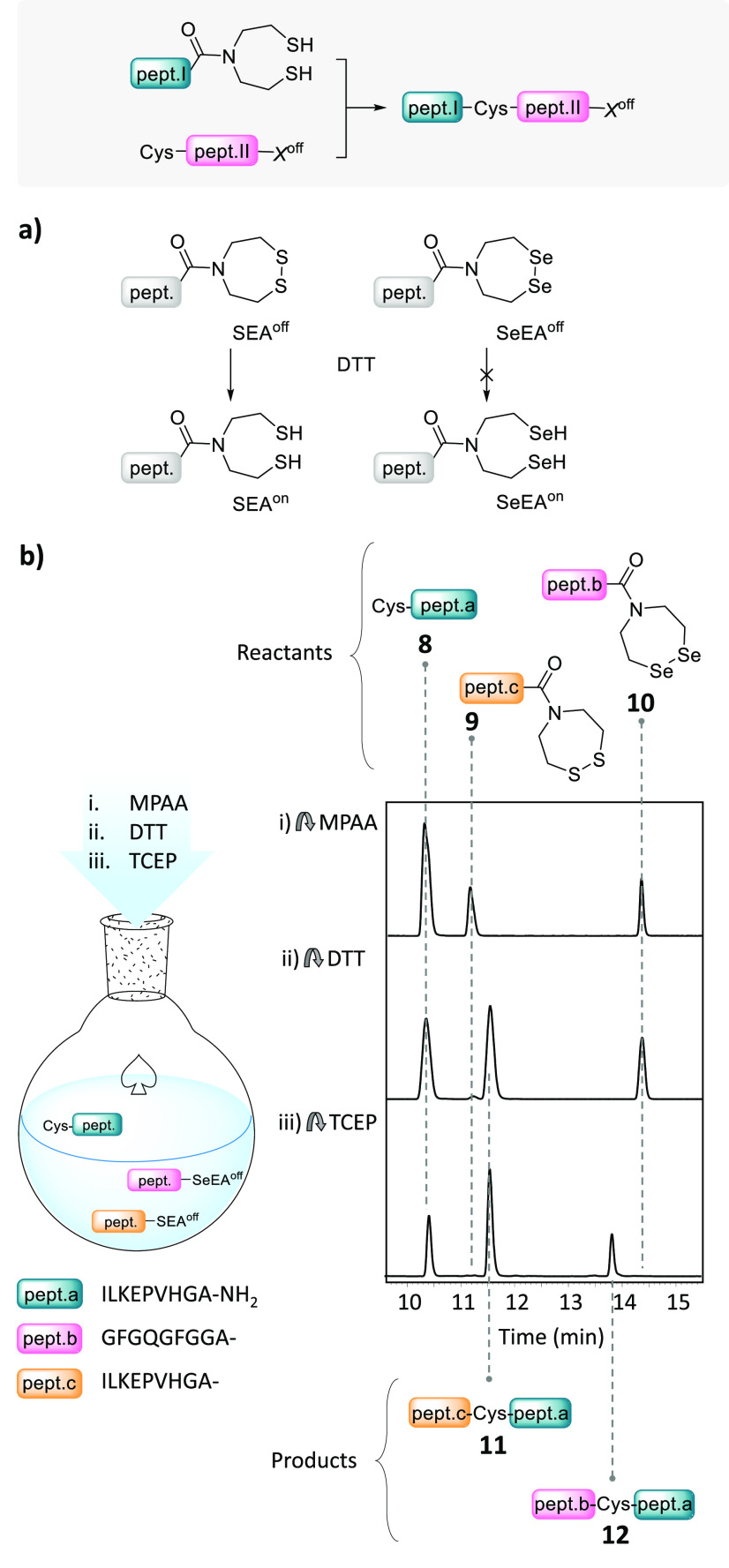
Design of the *bis*(2-selanylethyl)amido (SeEA) latent thioester. (a) The cyclic diselenide SeEA^off^ is stable toward MPAA and DTT. (b) Key experiment showing the selective and sequential activation of SEA^off^ and SeEA^off^ systems.

The extension of the concept depicted in Figure 2 to the assembly of four peptide segments in one-pot put us on the quest of a redox-controllable thioester system that can stay latent under the reducing conditions allowing SEA-mediated ligation (Figure 10). The nature of the R group attached to the SEA^off^ latent system is not expected to significantly change the redox properties of the cyclic disulfide or its accessibility because the chair conformation adopted by the 1,2,5-dithiazepane heterocycle projects the nitrogen substituent away from the disulfide bond (Figure 9b,c). Therefore, playing with the nature of the R group was not envisaged as a mean for diversifying the SEA^off^ family with respect to their sensitivity to reductants. An alternative would be to substitute the carbon atoms of the 1,2,5-dithiazepane scaffold, which are closer to the disulfide group, but at the expense of increased synthetic complexity.

A simple solution to this problem was achieved by substituting the sulfur atoms in the SEA^off^ group by selenium ones to place the acyl donor under the redox control of a cyclic diselenide bond (SeEA^off^, Figure 10a).^54^ By doing so, we hoped that the SeEA^off^ group would resist reduction and thus activation by DTT, a strong thiol reductant that we knew to be an alternative to TCEP for unlocking the SEA^off^ group. This idea was supported by the work of Iwaoka et al., who showed that the cyclic diselenide analogous to DTT cyclic disulfide could not be reduced by an excess of DTT.^55^ In practice, the SeEA^off^ group proved to be fully stable in the presence of MPAA and DTT, conditions that enable SEA-mediated ligation to proceed efficiently (formation of peptide **11**, Figure 10b). No trace of ligation product between SeEA^off^ peptide **10** and Cys peptide **8** could be detected by HPLC upon addition of DTT to the peptide mixture, even after prolonged reaction times. In contrast, the addition of TCEP in the mixture led to the reduction of the Se–Se bond and triggered the formation of ligation product **12** in high yield. The high selectivity achieved upon the activation of SEA^off^ and SeEA^off^ latent thioesters enabled us to assemble four peptide segments in one-pot through a sequential NCL/SEA/SeEA ligation process.^54^

**Figure 11:**
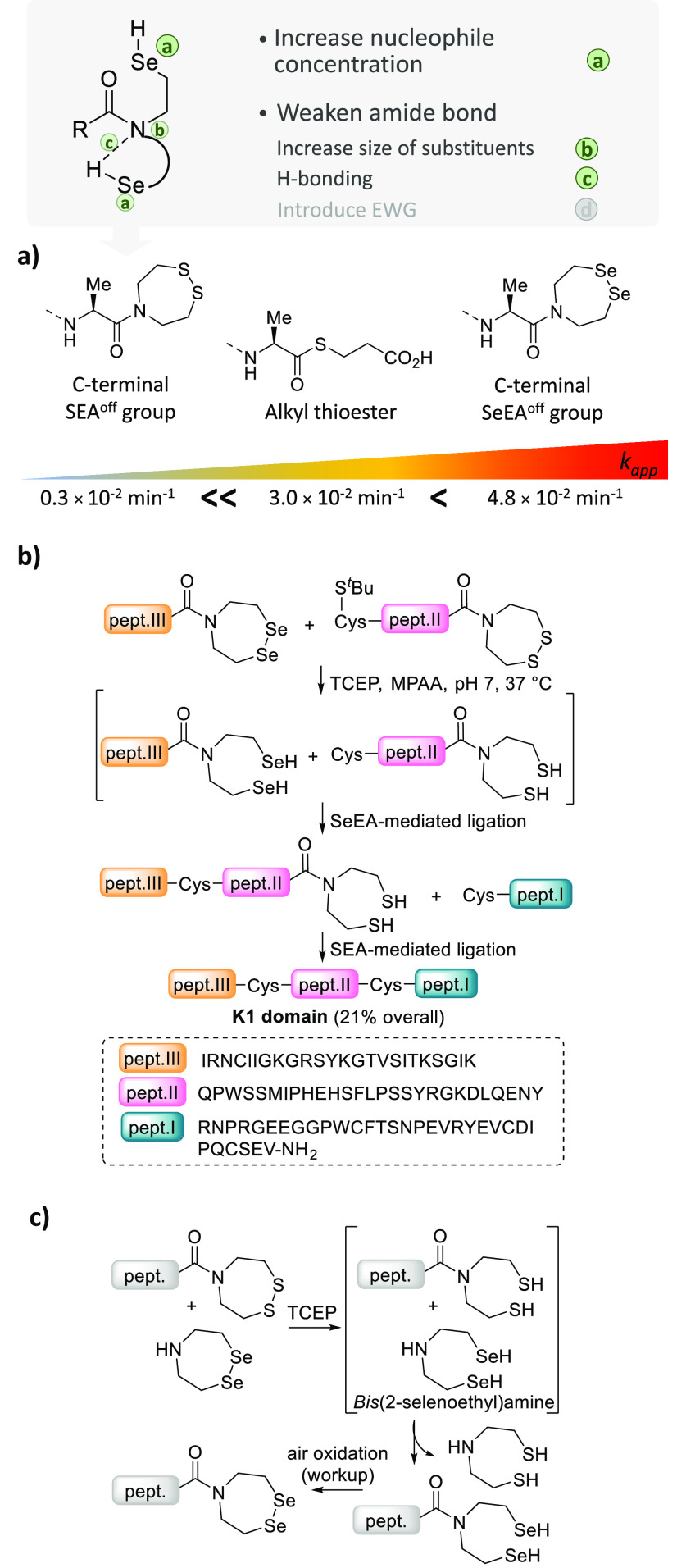
Impact of S → Se substitution on the acyl donor capability of the SEA/SeEA groups. (a) The kinetic data were obtained using Cys peptide CILKEPVHGV-NH_2_ at pH 7.2, 100 mM MPAA, 100 mM TCEP, and 37 °C (pseudo-first order rate law, data taken from ref (1)). (b) KCL of three peptide segments using SEA and SeEA-mediated ligations. (c) Synthesis of SeEA peptides.

In addition to this, we also established that the S → Se substitution in the SEA group offers another asset that is to significantly increase the rate of the ligation process on going from SEA to SeEA (Figure 11a).^1^ The more than 10-fold difference in the rate of SeEA and SEA-mediated ligations enables one to assemble three peptide segments in one-pot through a KCL approach (Figure 11b). Note that SeEA^off^ peptides are produced from SEA^off^ peptides by exchange with the *bis*(2-selenoethyl)amine (Figure 11c). An SPPS method for accessing SeEA peptides by Fmoc-SPPS is highly desirable and remains to be developed.

**Figure 12:**
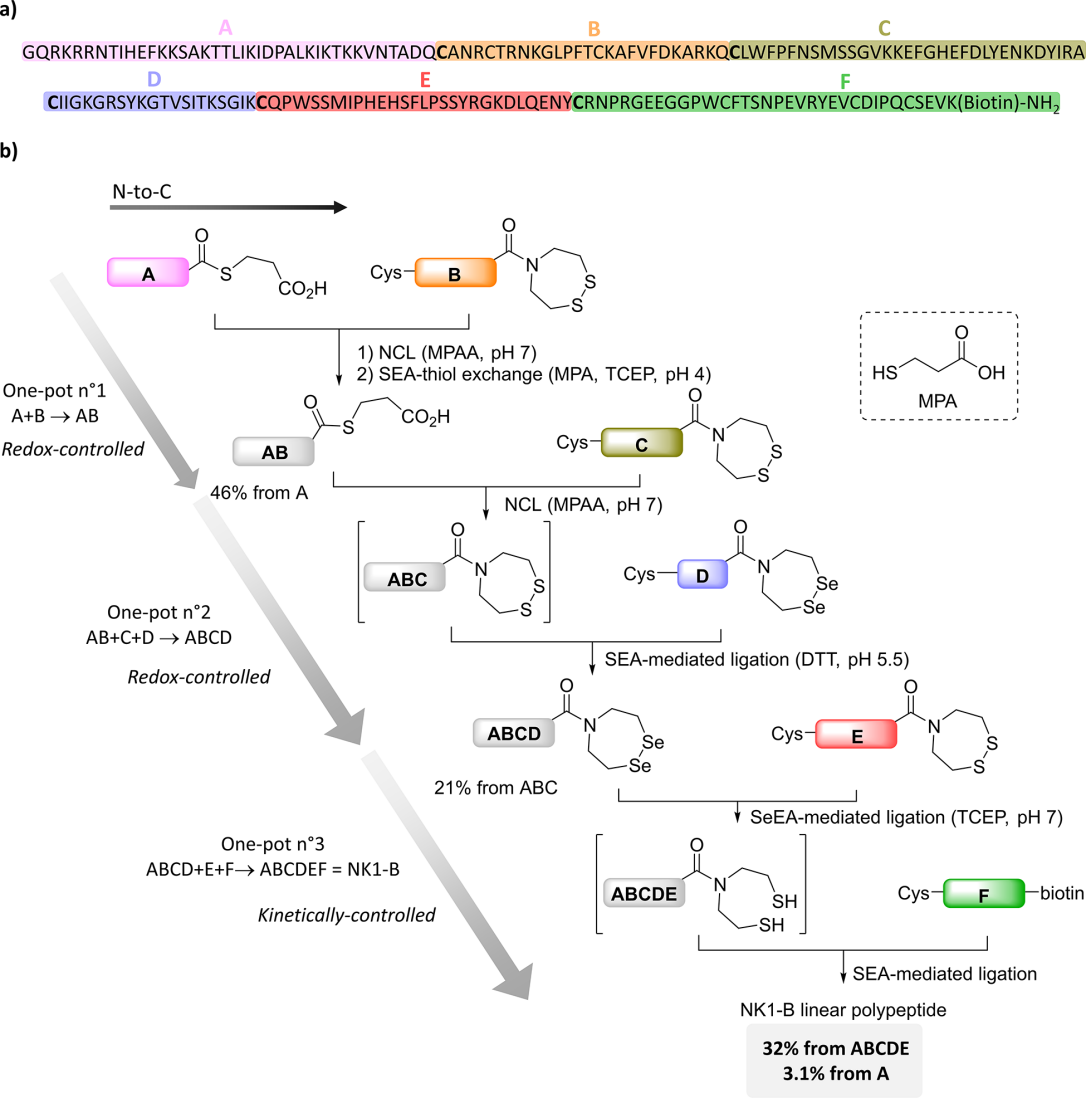
Chemical synthesis of NK1-B protein. (a) Sequence of NK1-B protein. (b) Total synthesis of biotinylated NK1 protein by combining SeEA/SEA-based redox- and kinetically-controlled assembly processes.

Although the SeEA/SEA-based KCL process is of great interest on its own, its value is heightened by the possibility of combining it with the SeEA/SEA redox-controlled approach presented before (Figure 10). This is possible because, in the redox-controlled assembly strategy utilizing SEA^off^ and SeEA^off^ functionalities, the SeEA^off^ group is unmasked in a late stage (Figure 10b), while it is activated at the beginning of the elongation in the KCL approach (Figure 11b). The connection of the above redox and KCL assembly processes enabled us to produce the 20 kDa biotinylated NK1 polypeptide NK1-B from the six peptide segments A–F (Figure 12). The first two one-pot processes were redox-controlled and yielded SeEA^off^ segment ABCD, which was isolated and subsequently engaged in a KCL process with segments E and F to complete the synthesis of NK1-B.

## 6 The Search for a Redox-Controlled Cys Surrogate

**Figure 13:**
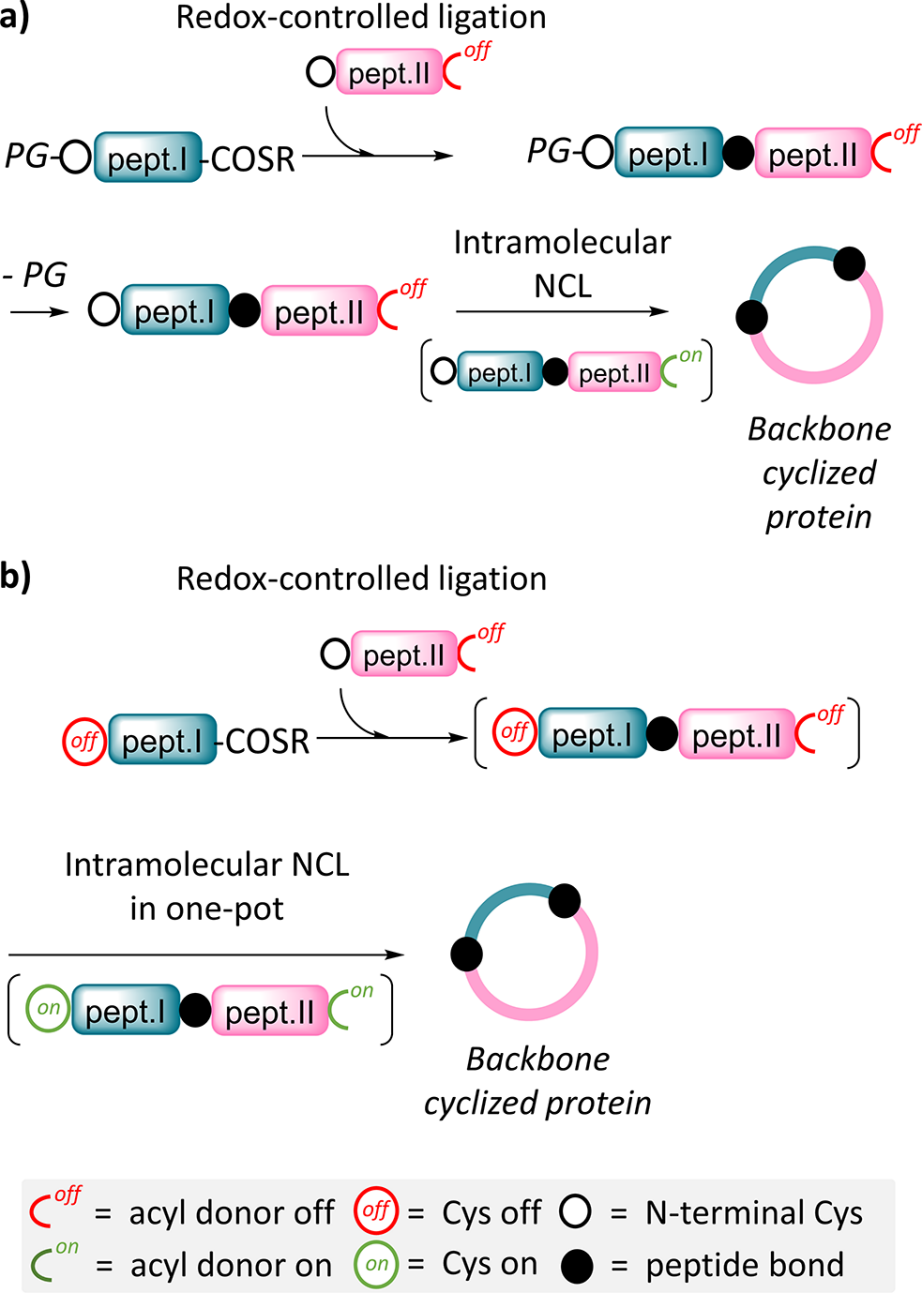
Access to sophisticated protein scaffolds such as cyclic proteins requires the late chemical manipulation of Cys residues. (a) Classical approach to the redox-controlled assembly of a cyclic protein using a protection strategy for the N-terminal Cys. (b) One-pot assembly of a cyclic protein using redox-controlled latent thioesters and Cys surrogates.

So far, we discussed the gain in pot-economy provided by the use of redox-controlled thioester surrogates in the chemical synthesis of proteins. In this regard, the synthesis of the NK1-B protein shows that a 20 kDa protein can be assembled through three successive one-pot processes without resorting to Cys PGs. Though always desirable, avoidance of the use of Cys PGs during SEA/SeEA-assisted protein synthesis is not always possible. Without dwelling on the rare cases when the primary sequence of the protein starts with a Cys residue, the resort to a Cys PG strategy cannot be avoided when a free Cys is required after the elongation step for further protein modification using the NCL reaction. The problem is illustrated in Figure 13a with the synthesis of a backbone cyclized protein from two peptide segments.

In recent years, significant progresses have been made to enable the deprotection of Cys in one-pot after NCL.^56,57^ On the forefront are the noble metal-assisted chemistries developed by Brik’s group.^57^ On our side, we searched for a redox-controlled Cys surrogate whose sensitivity to reductants could mirror that of SEA^off^, as such a combination might considerably simplify the access to sophisticated protein scaffolds in one-pot (Figure 13b). Though a simple idea, it took us years to find a solution to this problem, and we did so with the generous help of serendipity.

**Figure 14:**
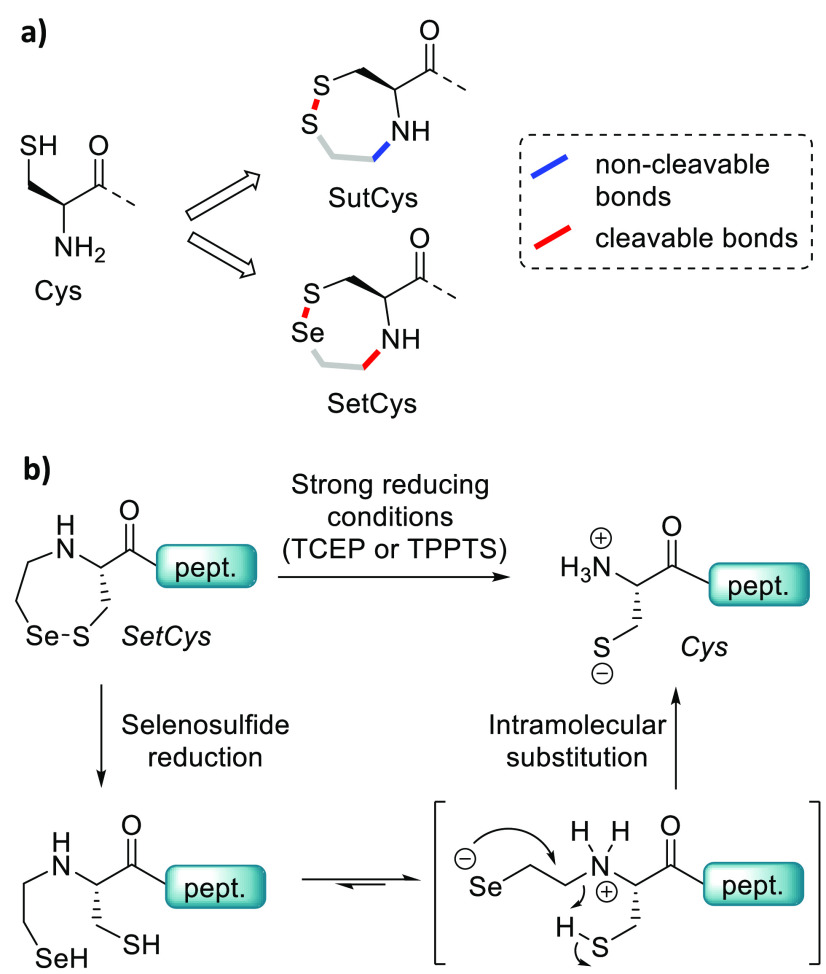
Conception of the SetCys redox-controlled cysteine surrogate. (a) Structure of SutCys and SetCys residues. (b) Proposed mechanism for the conversion of SetCys into Cys upon reduction.

The difficulty in designing a redox-controlled Cys surrogate comes from the lability and dynamic behavior of acyclic disulfides or selenosulfides derived from Cys thiol under classical NCL conditions. We noticed that simply appending a 2-mercaptoethyl limb to the α-amino group of cysteine results in a 1,5-dithiol structure that can form a 7-membered cyclic disulfide of the type we are looking for, i.e., SutCys in Figure 14a. Unfortunately, it can be easily predicted that such a Cys derivative can hardly be converted back to cysteine owing to the well-known difficulty in breaking a bond between an aliphatic carbon and a nitrogen atom,^3,58^ in line with previous studies on the *N*-(2-mercaptoethyl) auxiliary in the early days of NCL.^59^ With these considerations, we concluded that the Cys residue offers no obvious attachment point that could enable the design of a self-immolative cyclic disulfide.

The quest for a redox-sensitive Cys surrogate came again on stage when we discovered that the selenium analogue of SutCys called SetCys can spontaneously lose its selenoethyl arm upon reduction by TCEP or 3,3′,3″-phosphanetriyltris(benzenesulfonic acid) trisodium salt (TPPTS, Figure 14).^3,60^ We showed that the loss of the selenoethyl arm proceeds by an ionic mechanism whose rate is the fastest at pH 6–6.5, i.e., a pH compatible with NCL or SEA-mediated ligation. Our investigations strongly suggest that the cleavage of the C–N bond proceeds through an intramolecular substitution of the protonated SetCys alpha amino group by the selenoate as depicted in Figure 14b.

**Figure 15:**
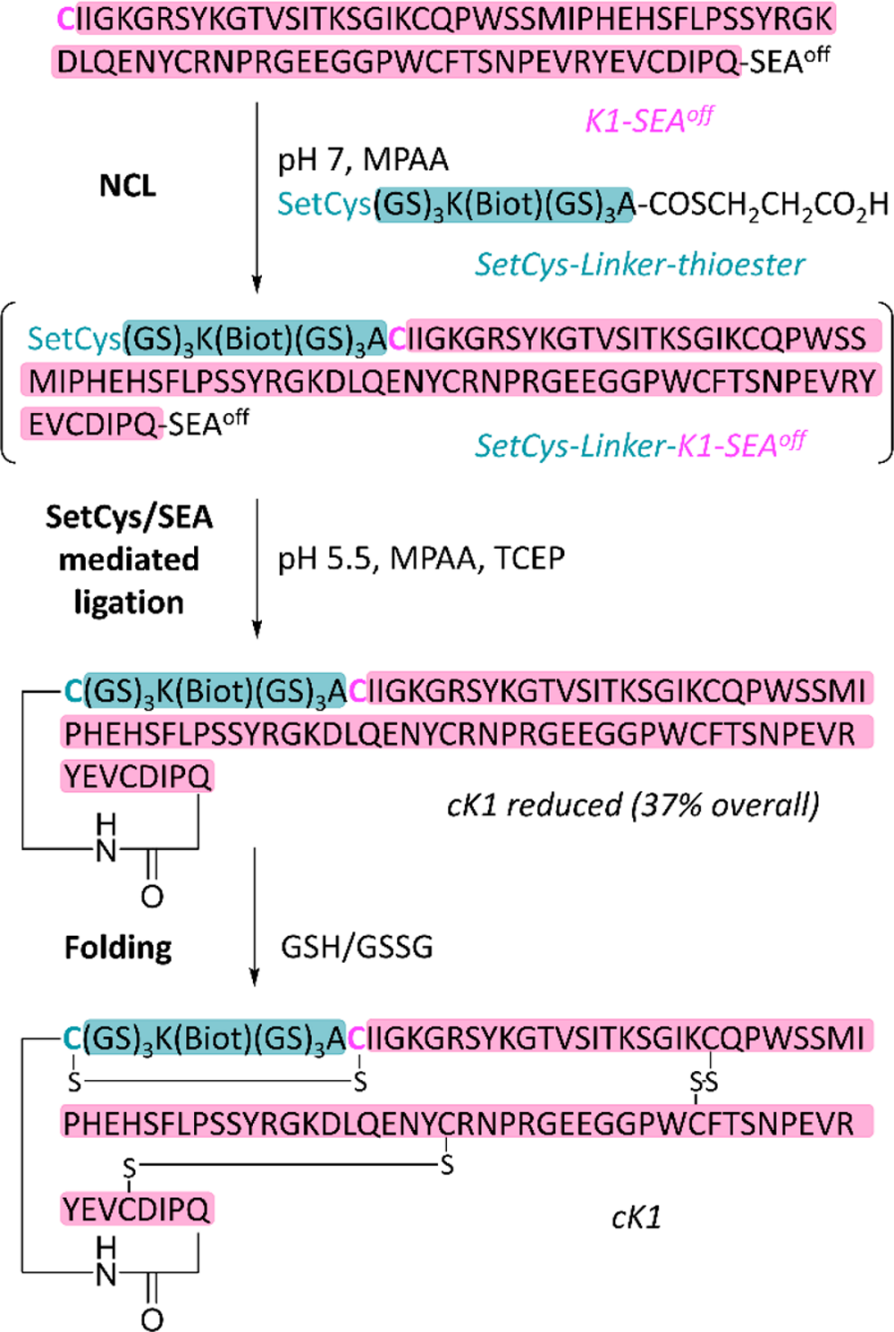
One-pot synthesis of a cyclic variant of the K1 HGF domain using a redox-controlled NCL-SetCys/SEA assembly process.

Fmoc-protected SetCys amino acid can be produced in gram scale from Cys. Introduced by Fmoc-SPPS in peptide segments, it comes to enlarge the tiny family of redox-controlled Cys surrogates, another interesting example being the α-azido cysteine that is activated by TCEP through a classical Staudinger reaction.^61^ As anticipated, SetCys can synergize with SEA^off^ latent thioester to access elaborated cyclic protein molecules using exactly the principle depicted in Figure 13b. In practice, the first step of such a process corresponds to a regular NCL reaction under weak reductive conditions (excess MPAA, Step 1, Figure 15). Under such conditions, the SetCys and SEA^off^ groups remain silent until TCEP is added to the mixture to trigger the second cyclative SetCys/SEA-mediated NCL process. The method was used to produce cyclic variants of the K1 HGF domain varying by the length of the linker joining K1 N- and C-termini. The backbone cyclized cK1 polypeptide was folded successfully and assayed for its agonistic activity on the MET receptor.

## 7 Conclusion and Outlook

The simplification of the chemical synthesis of a large variety of protein molecules is an important goal to pursue to make the chemical synthesis of proteins more widespread and easier to implement in the research lab as well as in industry for large scale production. The facilitation of the assembly of the polypeptide chain is one part of this big challenge. In this Account, we described our advances in the development of redox-controllable latent thioesters and cysteine surrogates. The structures of these chemical systems have in common the presence of a seven-membered ring cyclic dichalcogenide, the reduction of which triggers the ligation process. The placement of the formation of peptide bonds under the dependence of the reduction of disulfide, selenosulfide, or diselenide bonds provided the means to combine these systems with great selectivity just by experimenting with common and harmless reductants. One hallmark of the systems we have designed is the clustering of several O, N, S, and Se atoms in and around the seven-membered ring structure. This enabled us to achieve the desired properties by rendering C–N bonds spontaneously cleavable in water under mild conditions, while such a bond is usually very stable in a normal context. There is certainly more research to do in this direction to explore novel controllable reactions that can be set up with minimal synthetic efforts, cost, and waste.
